# Characterization of the planarian surface electroencephalogram

**DOI:** 10.1186/s12868-023-00799-z

**Published:** 2023-05-03

**Authors:** Jannes Freiberg, Lukas Lang, Christian Kaernbach, Julian Keil

**Affiliations:** Department of Psychology, Christian-Albrechts-University Kiel, Olshausenstrasse 62, 24118 Kiel, Germany

**Keywords:** Neural oscillation, 1/f law, Aperiodic component, Planarian, Animal cognition

## Abstract

**Background:**

Despite large morphological differences between the nervous systems of lower animals and humans, striking functional similarities have been reported. However, little is known about how these functional similarities translate to cognitive similarities. As a first step towards studying the cognitive abilities of simple nervous systems, we here characterize the ongoing electrophysiological activity of the planarian *Schmidtea mediterranea.* One previous report using invasive microelectrodes describes that the ongoing neural activity is characterized by a 1/f^x^ power spectrum with the exponent ‘x’ of the power spectrum close to 1. To extend these findings, we aimed to establish a recording protocol to measure ongoing neural activity safely and securely from alive and healthy planarians under different lighting conditions using non-invasive surface electrodes.

**Results:**

As a replication and extension of the previous results, we show that the ongoing neural activity is characterized by a 1/f^x^ power spectrum, that the exponent ‘x’ in living planarians is close to 1, and that changes in lighting induce changes in neural activity likely due to the planarian photophobia.

**Conclusions:**

We confirm the existence of continuous EEG activity in planarians and show that it is possible to noninvasively record this activity with surface wire electrodes. This opens up broad possibilities for continuous recordings across longer intervals, and repeated recordings from the same animals to study cognitive processes.

**Supplementary Information:**

The online version contains supplementary material available at 10.1186/s12868-023-00799-z.

## Background

Comparative cognitive science often strives to examine peak performance in different species [1]. To successfully understand the foundations of biological intelligence, it might be more constructive to examine the cognitive functions of lower animals [2]. This will then allow testing activity patterns related to stimulus processing and cognition, as well as the reproduction of these patterns in artificial neural networks. A first step into this direction is the characterization of the ongoing electrophysiological activity of such lower animals. Remarkably, Adrian [3] already recorded synchronized neural responses in the optic nerve of a water beetle. Despite large morphological differences between the nervous systems of lower animals and humans, striking similarities have since then become evident [4]. For example, honeybees show neural oscillations with a similar functional profile as the prominent human 10 Hz alpha oscillation [5]. Moreover, Aoki et al. [6] were able to record neural activity from the planarian flatworm *Schmidtea mediterranea.* Whereas these previous experiments were able to record a rich spectrum of neural activity, they involved decapitating the animals, opening the head capsule, or implanting invasive electrodes into the ganglia. To facilitate the recording of ongoing neural activity, ensure animal welfare and enable repeated recordings of the same animals, noninvasive recordings are necessary. The aim of the current experiment was to demonstrate the possibility to record neural activity safely and quickly from the planarian *Schmidtea mediterranea* without harming the animals.

The planarian *Schmidtea mediterranea*, formerly called *Dugesia mediterranea* is a small, approximately 20 mm long and 2 mm wide freshwater turbellarian of the order Tricladida, usually found around the Mediterranean Sea [7]. Planarians have successfully survived for 800 million years, and they are the closest living relatives to the original bilateralians, the first animals with two distinct hemispheres and a well-defined movement direction. In research, they are often used as model organisms due to their ability to regenerate the central nervous system (CNS) even from small pieces of the body within a short period of time [8]. This planarian is one of the simplest animals with a bilateral body plan and cephalization [9]. Its CNS itself comprises two lobed cephalic ganglia connected by the anterior commissure, which can be considered the most primitive brain in animal evolution. In addition, a pair of ventral nervous cords run the length of the animal, and both cords are connected by transverse commissures [10]. The cephalic ganglia appear to be remarkably complex, and using direct recordings from electrodes implanted in the ganglia Aoki and colleagues [6] were able to record ongoing neural activity characterized by a 1/f^x^ power spectrum with the exponent ‘x’ of the power spectrum close to 1. *Schmidtea mediterranea* can therefore be regarded as a primitive model organism for the human nervous system.

Whereas the CNS of *Schmidtea mediterranea* is comparatively simple, and resembles that of the early developmental stages of the CNS of vertebrates [11], the animal is nevertheless capable of complex behavior [12]. For example, the animal shows environmental familiarization, which persists across two weeks and even across regeneration of the cephalic ganglia [13]. Moreover, the planarians are mobile, avoid open spaces and are negatively phototactic [14], which promises at least basic cognitive capabilities such as sensory discrimination and decision making. While previous research showed ongoing neural activity, the cognitive ability to discriminate stimuli, and form long-term memory, it is currently unknown how these cognitive abilities are reflected in neural activity. As a first step towards shedding light on this issue, we here aimed to establish a recording protocol to record ongoing neural activity safely and securely from alive and healthy planarians under different lighting conditions. As a replication of the results by Aoki and colleagues [6], we hypothesized that the ongoing neural activity is characterized by a 1/f^x^ power spectrum with an exponent ‘x’ close to 1, and that changes in lighting will induce changes in neural activity due to the reported photophobia.

## Methods

The aim of the current experiment was to establish a recording protocol to noninvasively measure ongoing neural activity from live planarians without harming the animals. To this end, we developed a recording chamber and used a mobile electroencephalography (EEG) device to record the electrical signal from planarians in darkness, under light, and from dead mosquito larvae as a control group. To explore whether changes in neural activity under light stimulation are due to activation of the planarians’ eye spots, we also recorded electrical signals from decapitated animals. Please note that planarians fully regenerate after decapitation.

### Animal characteristics

Overall, we collected data from planarians of the asexual strain of the species *Schmidtea mediterranea*. Because we were unsure how the animals would respond to the experimental setting and to limit the stress on the individual animals, we chose a between-subjects design with 17 planarians recorded during darkness, and 19 recorded under illumination in the initial experiment. To examine, whether the changes in neural activity under light stimulation are due to the planarians’ response to light with their eye spots [15], we replicated the initial experiment with 20 decapitated planarians, of which 10 were recorded during darkness, and 10 were recorded under illumination. All planarians were bred for laboratory use and reared in our dedicated breeding facility at the Christian-Albrechts-University Kiel. Animals in the different groups were treated identically, fed daily, and all experiments took place between 11 a.m. and 3 p.m. As a control group to test how the recording setup responds to environmental noise, we recorded data from six red mosquito larvae purchased as food for our planarians. Mosquito larvae were used as a control group, because they were readily available in the laboratory, and because we were looking for an intact but dead animal with approximately the same size as a planarian.

### Recording environment

**Fig. 1:**
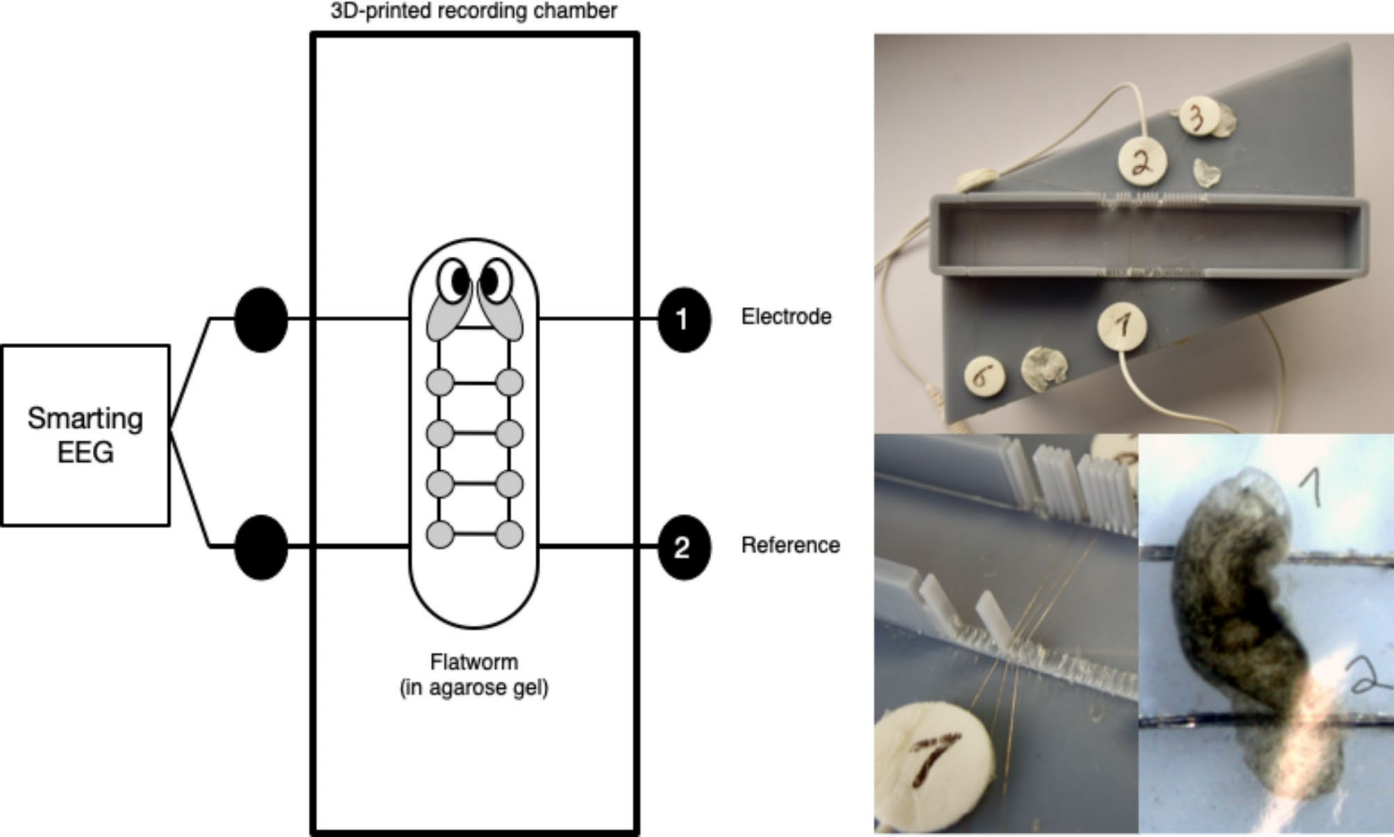
Schematic overview of the recording environment (left) and photograph of the device used in the experiments with one planarian on top of the electrodes (right). The animal is fixated in 2% agarose gel and placed on top of wire electroretinography electrodes. The electrodes are held in place in a 3D-printed recording chamber and are connected to a mobile EEG recording device

All recordings took place in the animal laboratories of the department of psychology at the Christian-Albrechts-University Kiel and were conducted by instructed student assistants supervised by specifically trained PhD students. In the first step, we designed and built a 3D-printed recording chamber to hold the electrodes and the planarians (Fig. 1). A key challenge was the choice of recording electrodes. Ring or cup electrodes used in human electroencephalography were too large for the planarians. Therefore, we chose wire electrodes, which are routinely used to record the electrical activity of the retina from the corneal surface [16], and which allow recordings of neural oscillations [17]. In the current setup, we used low impedance 65 mm long silver wire electroretinography electrodes (spesmedica DT0001, Italy). The ongoing electric activity was recorded from a mobile EEG amplifier (mBrainTrain Smarting, Serbia), and each electrode was referenced to the same reference electrode (bipolar montage). To immobilize the animals, they were placed on ice-cooled specimen slides, enclosed in 2% agarose gel (Roth, cooled down to 45 °C), and placed on top of the wire electrodes, so that the animals were directly touching the electrodes. The recording electrode was close to the head (or the decapitation site), and the reference electrode was close to the tail of the animal (Fig. 1). Please note that the mosquito larvae covered multiple recording electrodes, and these multiple electrodes were treated as independent recordings in the data analysis. The animals stayed on top of the electrodes for approximately 10 min, after which they were rinsed off to free them from the agarose gel.

### Stimulation protocol

The initial experiment comprised a between-subjects design with three groups. One group of planarians was recorded during darkness. The animals were enclosed in agarose gel and placed on the wire electrodes, afterwards the recording chamber as well as the EEG amplifier were covered with a mason tub (0 lx) and the EEG data were recorded for 10 min. The second group of planarians was recorded during light stimulation. As in the first group, the animals were enclosed in agarose gel and placed on the wire electrodes under ambient lighting. In this group, a light source (35 W Grow Light Bulb) 43 cm above the animals was switched on (approx. 40,000 lx), and the EEG data were recorded for 10 min. The surface temperature of the planarians was on average 18.97 °C (SD = 0.56 °C) before the light stimulation and 19.95 °C (SD = 1.21 °C) at the end of the experiment. The third group comprised dead mosquito larvae, which were placed on the wire electrodes without enclosing in agarose gel, and the EEG data were again recorded for 10 min. In the follow-up experiment, two groups of decapitated planarians were recorded in the identical recording environment as the first two groups in the initial experiment.

### Data processing

**Fig. 2:**
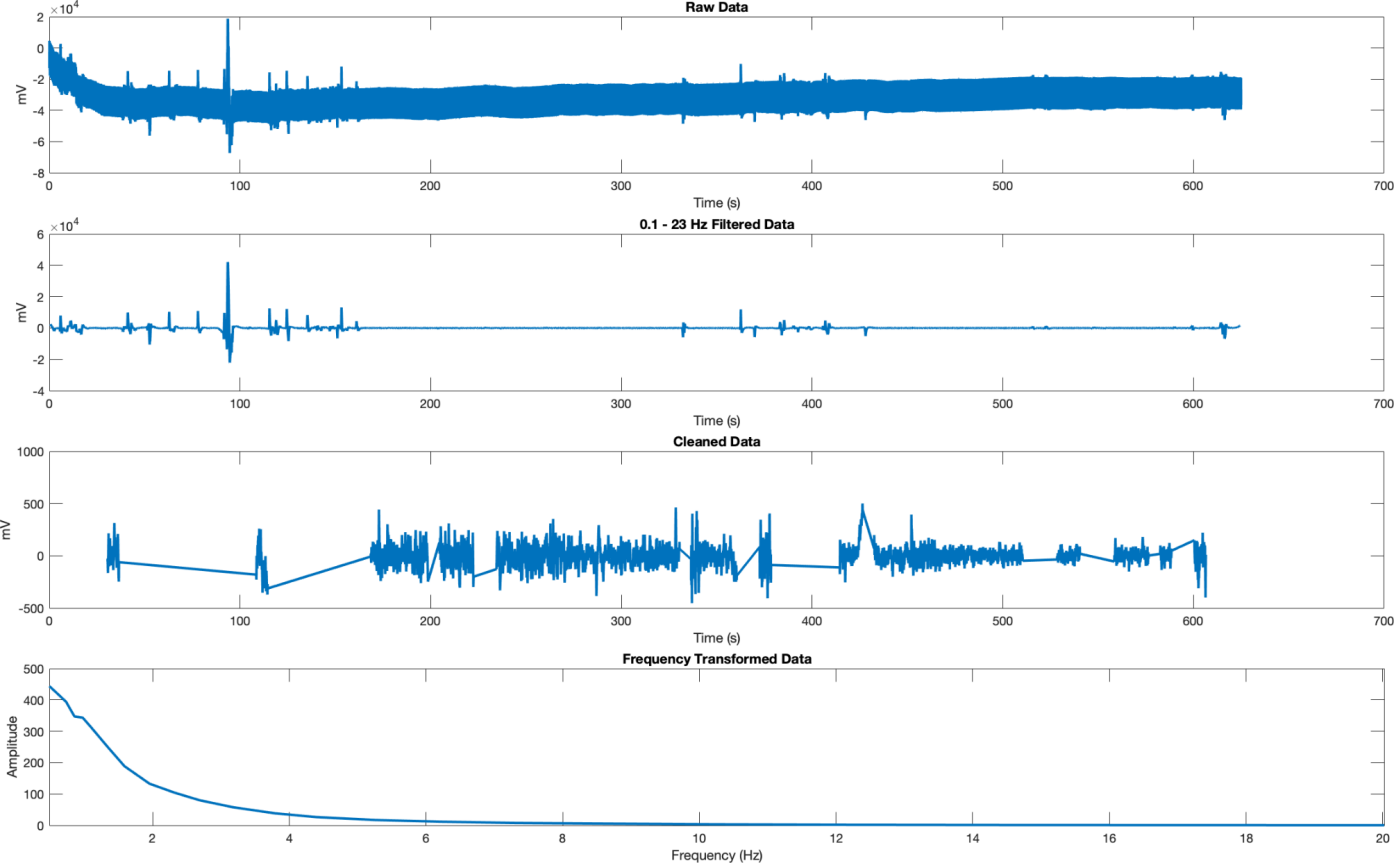
Overview of the data of one planarian recorded during darkness along different processing stages. Raw data was imported into Matlab. In the next step, the raw data were filtered between 0.1 and 23 Hz to remove line noise artifacts. Subsequently, the data were cut into 6 s-segments, and noisy segments were removed. Finally, data were transformed into the frequency domain between 0.5 and 20 Hz

In the current experiment, we recorded continuous EEG at a sample rate of 500 Hz at 24 bits resolution with a bandwidth of 0-250 Hz and one common reference electrode for 10 min. All raw data and data analysis scripts are available on OSF (https://osf.io/xf5cd/). All data analyses were performed in Matlab using the Fieldtrip toolbox [18] and custom-written code (https://github.com/juliankeil/VirtualTools). The entire data for each recording were imported into Matlab and filtered using two-pass Hamming-windowed FIR filters, with an order of 8250, a -6 dB cutoff frequency of 0.1 Hz, and a passband edge of 0.2 Hz for the high pass and an order of 288, a -6 dB cutoff frequency of 23 Hz, and a pass- band edge of 20.1 Hz for the low pass. The 23 Hz low-pass filter was chosen due to the high contamination with environmental noise at harmonic and subharmonic frequencies of the power line frequency. Moreover, Aoki et al. [6] report continuous spectra across the 0.1–5 Hz range. Subsequently, the first and last 500 ms of each dataset were removed to avoid edge artifacts, and the entire dataset was cut into non-overlapping 6 s segments. In order to automatically and reproducibly remove artifacts from the data, we then computed the mean signal amplitude for each channel and trial and removed channels and trials with peaks 5 standard deviations above or below the mean signal amplitude. Moreover, channels and trials were removed if they exceeded a threshold of 2.5 standard deviations above the mean variance, z-value, or kurtosis across channels and trials (using the “vt_autoreject” function). If at least one channel and more than 5 data segments survived the automatic artifact removal, spectral power and the exponent ‘x’ of the 1/f^x^ power spectrum (i.e., the so-called aperiodic component, [19]) were estimated using a multitaper fast Fourier transform with discrete prolate spheroidal sequence (DPSS) tapering with a spectral smoothing of ± 1 Hz (using the “ft_freqanalysis” function). The frequency band of 0.5–20 Hz was divided into 23 steps with logarithmic spacing (Fig. 2). Using this approach, we obtained 14 datasets for the first group of planarians recorded during darkness, 11 datasets for the second group of planarians recorded during light stimulation, and 18 datasets for the third group of dead mosquito larvae. Please note that the mosquito larvae covered multiple recording electrodes and we treated the single electrodes as independent recordings. In the follow-up experiment, 10 datasets were obtained for the first group of planarians recorded during darkness, and 10 datasets for the second group of planarians recorded during light stimulation. To examine the data at higher frequencies, we repeated the data analysis with dedicated notch filters at 25, 50 and 100 Hz, and examined the frequency space between 0.5 and 80 Hz in 44 steps with logarithmic spacing (Additional File 1).

### Data analysis

To differentiate neural activity between the three groups of the initial experiment, we assessed differences in oscillatory power and the exponent of the power spectrum between the two groups of planarians, and between each group of planarians and the control group of dead mosquito larvae (using the “ft_freqstatistics” function). To correct for multiple testing across the three comparisons, we adjusted the critical alpha-level to 0.01, and used the FDR correction for the correction for multiple comparisons across the different frequencies within each comparison. For each comparison of the power spectra, we conducted a nonparametric permutation test. The experimental test statistic was evaluated against a permutation (10,000 permutations) distribution to test the null hypothesis of no difference between the neural activity of the different groups using three two-tailed independent-samples tests. For each of the three comparisons of the exponents, we conducted two-tailed independent-samples t-tests.

The same data analysis approach was used in the follow-up experiment with decapitated planarians to examine the role of light perception from the eye spots, with the exception that four groups were compared here, i.e., the two groups of intact planarians of the initial experiment and the two groups of decapitated planarians, with one group recorded during light stimulation and one group record during darkness, respectively, in both experiments.

## Results

The aim of the current experiment was to record the electrical signal from planarians in darkness, under light, and from dead mosquito larvae as a control group, as well as from decapitated planarians. As a replication of the results by Aoki and colleagues [6], we hypothesized that the ongoing neural activity is characterized by a 1/f^x^ power spectrum, and that changes in lighting will induce changes in neural activity due to the reported photophobia.

### Power spectrum exponent

**Fig. 3:**
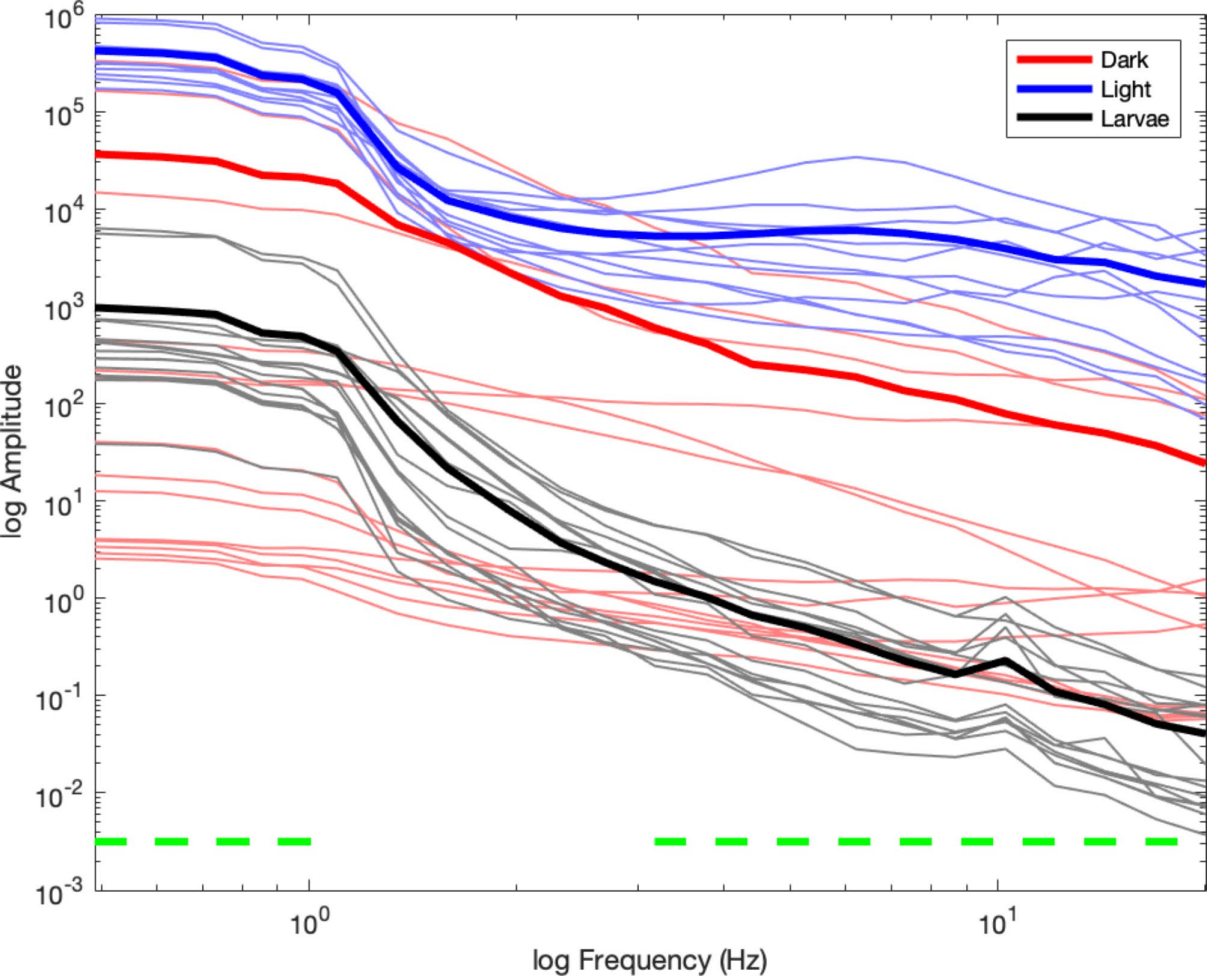
Overview of the power spectra for the three groups. The red line indicates the power spectral density for the first group of planarians recorded during darkness, the blue line indicates the power spectral density for the second group of planarians recorded during light stimulation, and the black line indicates the power spectral density for the third group of dead mosquito larvae recorded as a control group. Bold lines represent the mean across individuals, and the light lines represent the individual power spectra. The horizontal green lines indicate the frequency range in which the power spectra significantly differed between the first and second group

In a first step, we estimated the scaling exponent ‘x’ of the individual power spectra, which should follow a 1/f^x^ power law [20]. Larger exponents indicate steeper slopes, a value of 0 would indicate a flat slope, such as in white noise, and the exponent of human EEG is approximately 0.828 [19]. For the first group of intact planarians recorded during darkness, we found an average exponent of x = 1.23 (SD = 0.59), for the second group of intact planarians recorded during light stimulation, we found an average exponent of x = 1.31 (SD = 0.59), and for the dead mosquito larvae, we found an average exponent of x = 2.72 (SD = 0.22), as can be discerned from the comparison between power spectra in Fig. 3. Whereas the exponents did not differ between the two groups of intact planarians (t(21.61) = -0.35, p = 0.73, CI = [-0.57, 0.41]), it was significantly smaller for the darkness group compared to the mosquito larvae (t(15.78) = -8.99, p < 0.001, CI = [-1.83 -1.14]), and significantly smaller for the light stimulation group compared to the mosquito larvae (t(11.69) = -7.61, p < 0.001, CI = [-1.81 -1.00]). In the follow-up experiment with decapitated planarians, we found an average exponent of x = 1.32 (SD = 1.16) during darkness, and x = 1.48 (SD = 0.93) during light stimulation. These values neither differed within the decapitated group (t(17.15) = -0.33, p = 0.75, CI = [-1.15 0.84]), nor within the darkness group (t(12.29) = -0.24, p = 0.81, CI = [-0.97 0.77]), or the light stimulation group (t(14.98) = -0.49, p = 0.63, CI = [-0.89 0.56]).

### Oscillatory activity

In the second step, we estimated the power of oscillatory activity between 0.5 and 20 Hz with logarithmic frequency spacing. The permutation-based comparison between groups with FDR correction for multiple comparisons indicated that the power of oscillatory activity differed between the planarians recorded during darkness and under light stimulation in a low frequency range between 0.488 and 1.098 Hz and in a higher frequency range between 3.174 and 20.019 Hz (see Table 1 for details), as indicated by the green horizontal lines in Fig. 3. The power spectra from the planarians under light stimulation differed across the entire frequency range from the spectra recorded from the dead mosquito larvae. However, only the power above 7.324 Hz differed between the planarians recorded during darkness and the dead mosquito larvae.

**Table 1:** Statistical results for the comparison of oscillatory power between 0.5 and 20 Hz for the three groups (planarians recorded during darkness and under light stimulation, and dead mosquito larvae). T- and p-values result from permutation-based between-groups comparisons, and significance thresholds are FDR corrected for multiple comparisons

Freq.	0.488	0.610	0.732	0.854	0.976	1.098	1.342	1.586	1.953	2.319	2.685	3.173	3.784	4.394	5.249	6.225	7.324	8.666	10.253	12.084	14.282	16.967	20.019
**Dark vs. Light**																							
**t-Val.**	-5.5112	-5.4146	-5.3160	-5.3980	-5.3536	-5.2769	-2.6863	-1.5758	-2.2749	-3.1463	-3.3386	-3.6734	-3.3596	-3.0185	-2.5209	-2.2532	-2.3810	-2.8230	-3.1953	-3.3866	-3.5259	-3.4452	-3.1491
**p-Val.**	0.0001	0.0001	0.0001	0.0001	0.0001	0.0001	0.0050	0.0831	0.0168	0.0035	0.0027	0.0007	0.0001	0.0001	0.0001	0.0001	0.0001	0.0001	0.0001	0.0001	0.0001	0.0001	0.0001
**Sig.**	**true**	**true**	**true**	**true**	**true**	**true**	false	false	false	false	false	**true**	**true**	**true**	**true**	**true**	**true**	**true**	**true**	**true**	**true**	**true**	**true**
**Dark vs. mosquito**																							
**t-Val.**	1.5881	1.5834	1.5876	1.5703	1.5612	1.5280	1.4319	1.3781	1.3921	1.4357	1.4121	1.4968	1.5725	1.7907	1.7342	1.7045	1.7685	1.8448	1.9822	2.0371	2.1290	2.2370	2.3449
**p-Val.**	0.0707	0.0718	0.0740	0.0697	0.0695	0.0686	0.0475	0.0139	0.0032	0.0023	0.0023	0.0020	0.0018	0.0012	0.0007	0.0005	0.0003	0.0002	0.0003	0.0001	0.0001	0.0001	0.0001
**Sig.**	false	false	false	false	false	false	false	false	false	false	false	false	false	false	false	false	**true**	**true**	**true**	**true**	**true**	**true**	**true**
**Light vs. mosquito**																							
**t-Val.**	7.5388	7.3699	7.2229	7.6010	7.6667	8.3619	7.4187	5.5510	5.8444	6.3308	5.9535	5.1780	4.2716	3.6281	2.9948	2.6566	2.7857	3.2985	3.7224	3.9456	4.0969	4.0049	3.6454
**p-Val.**	0.0001	0.0001	0.0001	0.0001	0.0001	0.0001	0.0001	0.0001	0.0001	0.0001	0.0001	0.0001	0.0001	0.0001	0.0001	0.0001	0.0001	0.0001	0.0001	0.0001	0.0001	0.0001	0.0001
**Sig.**	**true**	**true**	**true**	**true**	**true**	**true**	**true**	**true**	**true**	**true**	**true**	**true**	**true**	**true**	**true**	**true**	**true**	**true**	**true**	**true**	**true**	**true**	**true**

**Fig. 4:**
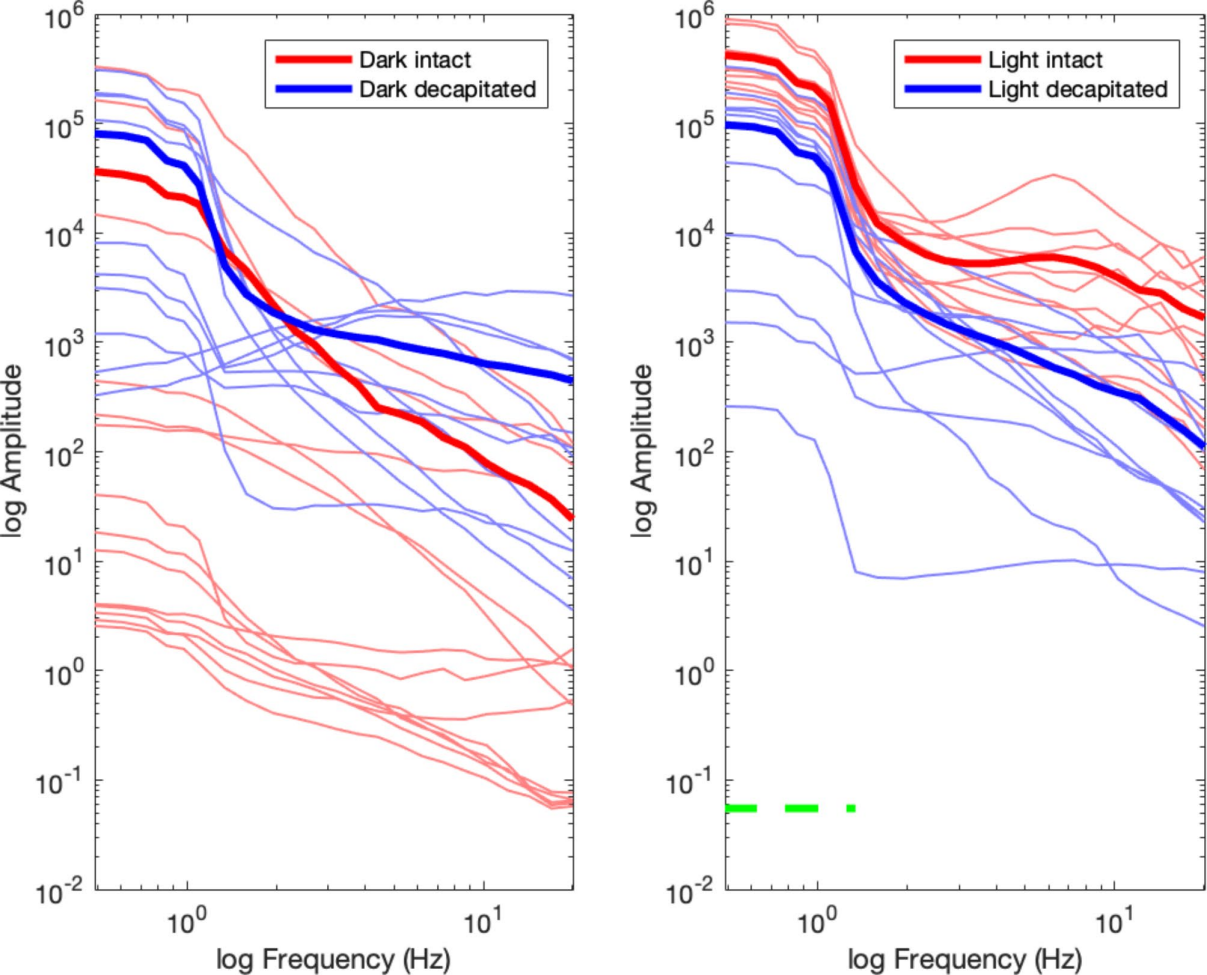
Overview of the power spectra for the follow-up experiment for recording during darkness (left) and under light stimulation (right). The red line indicates the power spectral density for the intact group of planarians, and the blue line indicates the power spectral density for the group of decapitated planarians. Bold lines represent the mean across individuals, and the light lines represent the individual power spectra. The horizontal green lines indicate the frequency range in which the power spectra significantly differed between the intact and decapitated animals

In the follow-up experiment, we found that the power of oscillatory activity recorded from decapitated planarians during darkness and under light stimulation did not differ across the entire frequency range (see Table 2 for details). Similarly, during darkness, the power spectra of intact and decapitated planarians did not differ. In contrast, under light stimulation, low-frequency power between 0.488 and 1.342 Hz was higher for the intact than for the decapitated animals, as indicated by the green horizontal line in Fig. 4, which suggests that the eye-spots are necessary for the perception of light.

**Table 2:** Statistical results for the comparison of oscillatory power between 0.5 and 20 Hz for the follow-up experiment (intact and decapitated planarians recorded during darkness and under light stimulation). T- and p-values result from permutation-based between-groups comparisons, and significance thresholds are FDR corrected for multiple comparisons

Freq.	0.488	0.610	0.732	0.854	0.976	1.098	1.342	1.586	1.953	2.319	2.685	3.173	3.784	4.394	5.249	6.225	7.324	8.666	10.253	12.084	14.282	16.967	20.019
**Dark decapitated vs. Light decapitated**																							
**t-Val.**	-0.3400	-0.3335	-0.3189	-0.3230	-0.3540	-0.3939	-0.5300	-0.4279	-0.3421	-0.2638	-0.2284	-0.0777	0.1011	0.3115	0.5122	0.7397	0.8318	0.9222	0.9165	0.9035	1.0829	1.1914	1.2515
**p-Val.**	0.3695	0.3716	0.3770	0.3700	0.3599	0.3511	0.3045	0.3588	0.3702	0.3899	0.3950	0.4657	0.4568	0.3740	0.3020	0.2333	0.2109	0.1994	0.2053	0.2250	0.1720	0.1392	0.1034
**Sig.**	false	false	false	false	false	false	false	false	false	false	false	false	false	false	false	false	false	false	false	false	false	false	false
**Dark intact vs. Dark decapitated**																							
**t-Val.**	-1.0471	-1.0817	-1.0871	-0.9534	-0.8713	-0.5030	0.2780	0.3841	0.1480	-0.1951	-0.3261	-0.8639	-1.4261	-2.2101	-2.2611	-2.3073	-2.4533	-2.2756	-2.2980	-2.1506	-2.0511	-1.9723	-1.9165
**p-Val.**	0.1653	0.1516	0.1492	0.1880	0.2001	0.3283	0.4795	0.5000	0.4582	0.4274	0.3938	0.1778	0.0952	0.0214	0.0159	0.0121	0.0083	0.0135	0.0110	0.0105	0.0109	0.0092	0.0052
**Sig.**	false	false	false	false	false	false	false	false	false	false	false	false	false	false	false	false	false	false	false	false	false	false	false
**Light intact vs. Light decapitated**																							
**t-Val.**	3.9224	3.8292	3.7659	3.9538	3.9735	4.3138	3.7729	2.7284	2.8534	3.0726	3.0081	2.7923	2.4612	2.2063	1.9223	1.7577	1.8583	2.2166	2.4821	2.5855	2.7622	2.7009	2.4939
**p-Val.**	0.0002	0.0002	0.0002	0.0002	0.0002	0.0002	0.0003	0.0009	0.0031	0.0026	0.0026	0.0027	0.0022	0.0020	0.0013	0.0009	0.0010	0.0008	0.0012	0.0013	0.0011	0.0014	0.0021
**Sig.**	**true**	**true**	**true**	**true**	**true**	**true**	**true**	false	false	false	false	false	false	false	false	false	false	false	false	false	false	false	false

We conducted the same analysis on the data in the frequency range between 0.5 and 80 Hz, and the results remained qualitatively similar. However, the frequency range above the first powerline subharmonic was heavily contaminated by noise (Additional File 1).

## Discussion

A large part of the field of comparative cognitive science aims to examine peak performance across different species [1]. An alternative approach is searching for the most primitive nervous system capable of a certain cognitive task such as memory or sensory discrimination. Here, we studied *Schmidtea mediterranea*, a small planarian with only 50,000 neurons capable of astounding cognitive tasks, in an attempt to pin down neural activity corresponding to stimulus processing in a relatively simple nervous system. A previous study [6] used invasive monopole recordings in cooled planarians, and observed ongoing activity between 0.1 and 5 Hz with a power spectrum characterized by a 1/f^1^ relationship. Our main goal of this research project was establishing a recording protocol to record ongoing neural activity safely and securely from alive planarians under different lighting conditions. As a replication of the previous results, we hypothesized that the ongoing neural activity is characterized by a 1/f^1^ power spectrum, and that changes in lighting will induce changes in neural activity likely due to the reported photophobia.

### Power spectrum exponent

Studying neural oscillations and their relationship to cognitive processes is a hallmark of cognitive neuroscience [21]. However, neural oscillations, defined as rhythmic activity within a narrow frequency band, are embedded in aperiodic activity [19]. This aperiodic part of the neural power spectrum has been related to the integration of excitatory and inhibitory synaptic currents [22] [20]. The power spectral density of the aperiodic component of neural data follows a 1/f^x^ power law and the exponent ‘x’ describes how steep or flat the power spectral density is [23]. Larger values of x indicate steeper slopes, a value of 0 would indicate a flat slope, such as in white noise. The smaller the exponent the larger the excitation to inhibition ratio. Accordingly, in a vigilant state the exponent is smaller than in unconscious states which indicates more neural excitation [24]. The exponent of human EEG is approximately 0.828 [19], and the planarian EEG previously showed good correspondence with an exponent of 1 [6]. Here, we observed that the exponent of the planarian EEG is 1.31 during darkness and 1.23 during light stimulation (1.48 and 1.32 in decapitated planarians), indicating a slightly steeper slope compared to humans and previous planarian EEG recordings, but still within the expected range of biological neural networks (cf., Fig. 5e in [19] which indicates an exponent range of approximately 1 to 1.75 for younger participants). The current results also closely resemble Fig. 5 in [6] with a reference line for an exponent of 1 (Additional File 2). Importantly, we observed a vastly steeper slope from the recordings of the dead mosquito larvae. We can therefore conclude that we were successful in recording neural activity from planarians with our current setup, because the exponent of the power spectrum resembles the exponents observed in recordings of neural activity from planarian and human EEG and differs significantly from the exponent extracted from recordings of dead animals. However, the steep slope extracted in the latter recordings indicates the presence of low frequency noise in our recording setup, which we will need to address in future experiments.

### Oscillatory activity

In addition to the aperiodic component of the spectrum of neural activity, periodic parameters like frequency and power can change with cognitive tasks or different stimulation conditions [25]. A vast amount of research across species has related different frequency bands of neural activity to different behaviors, perceptual or cognitive processes, or conscious states [26] [27]. Much of this research has focused on prominent peaks of neural activity visible in the power spectrum of neural activity, such as the 10 Hz alpha rhythm in humans related to attention [28] [29], the 6 Hz theta rhythm in rodents related to memory [30] [31], or the higher beta rhythm between 15 and 35 Hz related to perceptual decision making or motor activity [32] [33]. In planarians, the previous work by Aoki and colleagues [6] did not uncover specific peaks in the power spectrum, aside from a “plateau” between 0.1 and 0.4 Hz. Similarly, the data recorded in our experiment under darkness did not show any specific peaks aside from a plateau in lower frequencies. However, the low-frequency signal was not significantly different from the signal recorded from the dead mosquito larvae, so it is possible that this plateau reflects noise in the recording setup. In contrast, in the comparison of the power spectra between the planarians recorded during darkness and under light stimulation, we found that the overall power appears to be increased. More specifically, we found a significant difference at low frequencies between approximately 0.5 and 1 Hz, indicating an elevated low-frequency plateau during light stimulation. In addition, we also found a significant difference in a broad frequency range between 3 and 20 Hz, indicating increased higher frequency power during light stimulation. Importantly, in the decapitated planarians, we found an absence of the signal increase due to light stimulation, and only the low-frequency power between 0.5 and 1.3 Hz was significantly increased in the intact compared to the decapitated planarians. Thus, the latter activity could be specifically related to the reported photophobia of the planarians. Aoki et al. [6] reported increased waveform activity when the animals were warmed to approximately 10 °C, as well as large myogenic spikes. Our data processing pipeline should have excluded all myogenic spikes due to their large amplitude above the signal mean (Fig. 2). Therefore, we can conclude that the difference in the power spectra likely reflects increased neural activity due to sensory stimulation or to an overall active state of the live, uncooled animals. The exact nature of the low- and high-frequency power increase needs to be further examined in different stimulation contexts or in different tasks.

### Limitations

Previous research demonstrated the possibility to record neural activity from the planarian cephalic ganglia using invasive monopole electrodes [6]. We extend this demonstration by recording ongoing electrophysiological activity from noninvasive surface electrodes at room temperature. Whereas this approach has several advantages over the invasive recordings, such as reducing the harm to the animals, ease and low cost of implementation of the recording procedure, and the possibility to record the same animal multiple times, it comes with a number of drawbacks. First, the recording device is not specifically designed to record from planarians, and it is imperative to carefully establish that the currently observed signal reflects neural activity. To this end, we recorded EEG activity with the same setup from dead mosquito larvae. Second, and related to this, our recording setup is spatially much less precise than a single electrode inserted into the cephalic ganglion. Therefore, future recording setups should include a higher number of electrodes to disentangle electrical activity arising from the ganglia from those arising from the ventral nervous cords, or muscle activity. Finally, we observed strong low-frequency noise even in the recordings from the mosquito larvae, and strong signal contamination from the power lines, even at sub-harmonic frequencies (Additional File 1). Therefore, we need to optimize our current recording environment to shield it from environmental noise, for example with a Faraday cage. Whereas we show changes in the power spectrum between darkness and light stimulation, other stimulations should be considered, such as vibrations [6]. Moreover, recent advances in RNA injection suggest that it is possible to specifically remove eye spots or block UV light responsiveness [34] [15]. These manipulations could further help determining whether the recorded signals reflect neural activity.

## Conclusion

Overall, we can confirm the observations by Aoki and colleagues [6], that the power spectrum of continuous neural activity of planarians exhibits a broad rise in the low frequencies, and that the spectrum conforms to a 1/f^x^ pattern with an exponent ‘x’ close to 1. Moreover, we extend this observation by showing that light stimulation induced an increase in higher frequency activity. Recording neural activity from planarians with wire electrodes allows continuous recordings across longer intervals, and repeated recordings from the same animals without harming the animals to study changes in neural activity linked to stimulus processing and cognition. Studying nervous systems with a small number of neurons to understand the foundations of biological intelligence is an interesting interdisciplinary bridge to the field of artificial intelligence. Simulations and hardware implementations of artificial neural networks strive to disentangle the mechanisms underlying different cognitive performances such as memory or perception. It would be highly interesting to compare biological and artificial neural networks with similar properties to quantify the minimal number of neurons necessary for a certain task.

## Electronic supplementary material

Below is the link to the electronic supplementary material.

Supplementary Material 1

Supplementary Material 2

## Data Availability

The datasets generated and analyzed during the current study are available in at https://osf.io/xf5cd/.
