# Supplementary material for: Characterization of the planarian surface electroencephalogram
Source: BMC Neurosci. 2023 May 3;24:29. doi: 10.1186/s12868-023-00799-z (PMC10157967; doi:10.1186/s12868-023-00799-z)
Supplement: Supplementary file 1 — Supplementary Material 1 [file 12868_2023_799_MOESM1_ESM.pdf]

Additional File 1

Data Analysis with dedicated notch filters at harmonics of the power line noise

The raw data obtained from recording planarians using wire electrodes is heavily contaminated with power line noise at 50 Hz, subharmonic noise at 25 Hz, and harmonic noise at 100 Hz. Therefore, and in line with previous reports on the planarian electrophysiology we initially decided to focus on the low frequency range below 20 Hz. However, in order to fully appreciate the neural activity, we repeated the data analysis with a high-pass FIR filter at 0.1 Hz (order = 8250, -6 dB cutoff frequency = 0.1 Hz, passband edge = 0.2 Hz), low-pass FIR filter at 95 Hz (order = 70, -6 dB cutoff frequency = 95 Hz, passband edge = 83.1 Hz), and dedicated notch filters at 24 to 26 Hz (order = 826, -6 dB cutoff frequencies = [24 26] Hz, stopband edge = 25 Hz, passband edge = 27 Hz) and at 48 to 52 Hz (order = 414, -6 dB cutoff frequencies = [48 52] Hz, stopband edge = 50 Hz, passband edge = 54 Hz). Subsequently, spectral power was estimated using a multitaper fast Fourier transform with discrete prolate spheroidal sequence (DPSS) tapering with a spectral smoothing of ±1 Hz (using the “ft_freqanalysis” function). The frequency band of 0.5–80 Hz was divided into 44 steps with logarithmic spacing.

The results obtained from the analysis with a broader frequency range were qualitatively similar to the initial analysis focused on frequencies below 50 Hz. The permutation-based comparison between groups with FDR correction for multiple comparisons indicated that the power of oscillatory activity differed between the planarians recorded during darkness and under light stimulation in a low frequency range between 0.488 and 1.2207 Hz and in a higher frequency range between 2.6855 and 36.3770 Hz, with an additional high-frequency difference at 71.4111 Hz, as indicated by the green horizontal lines in figure A1.

Whereas the results from the data analysis remained qualitatively similar to the initial data analysis on the frequencies below 20 Hz, it becomes clear from the power spectra that the data is heavily contaminated with noise despite the dedicated notch frequencies. Further experiments using similar setups should therefore pay close attention to avoiding external noise by for example using a faraday cage around the recording setup.

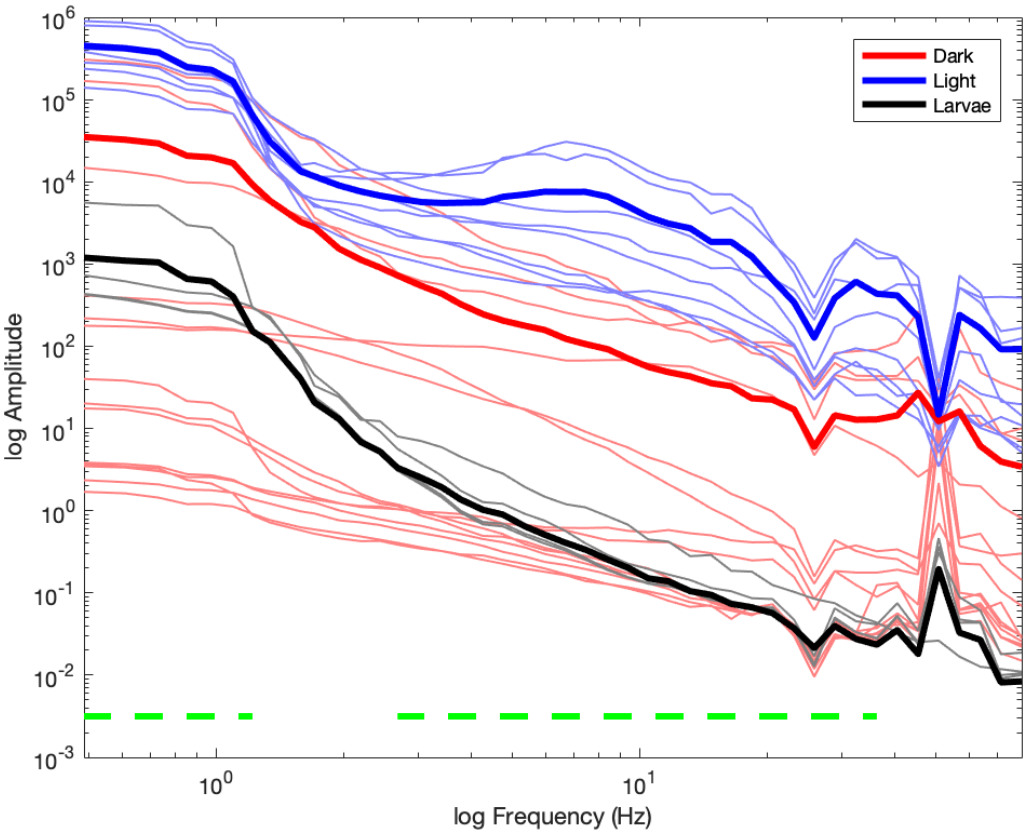

Figure A1: Overview of the power spectra for the three groups. The red line indicates the power spectral density for the first group of planarians recorded during darkness, the blue line indicates the power spectral density for the second group of planarians recorded during light stimulation, and the black line indicates the power spectral density for the third group of dead mosquito larvae recorded as a control group. Bold lines represent the mean across individuals, and the light lines represent the individual power spectra. The horizontal green lines indicate the frequency range in which the power spectra significantly differed between the first and second group.
