# Supplementary material for: Characterization of the planarian surface electroencephalogram
Source: BMC Neurosci. 2023 May 3;24:29. doi: 10.1186/s12868-023-00799-z (PMC10157967; doi:10.1186/s12868-023-00799-z)
Supplement: Supplementary file 2 — Supplementary Material 2 [file 12868_2023_799_MOESM2_ESM.pdf]

Additional File 1

Data Analysis with dedicated notch filters at harmonics of the power line noise

The power spectral density of the aperiodic component of neural data follows a $1/f^x$ power law and the exponent ‘x’ describes how steep or flat the power spectral density is. In their analysis of microelectrode recordings from the cerebral ganglia of planarians, Aoki and colleagues described a good correspondence of the power spectral density to a linear decrease of power with increasing frequency with a coefficient of 1. In the current analysis of data record from planarians at rest during darkness, we found an exponent of x = 1.31. From the direct comparison between the power spectra obtained by Aoki an colleagues and the current data in figure A2, the resemblance of the power spectra becomes clear.

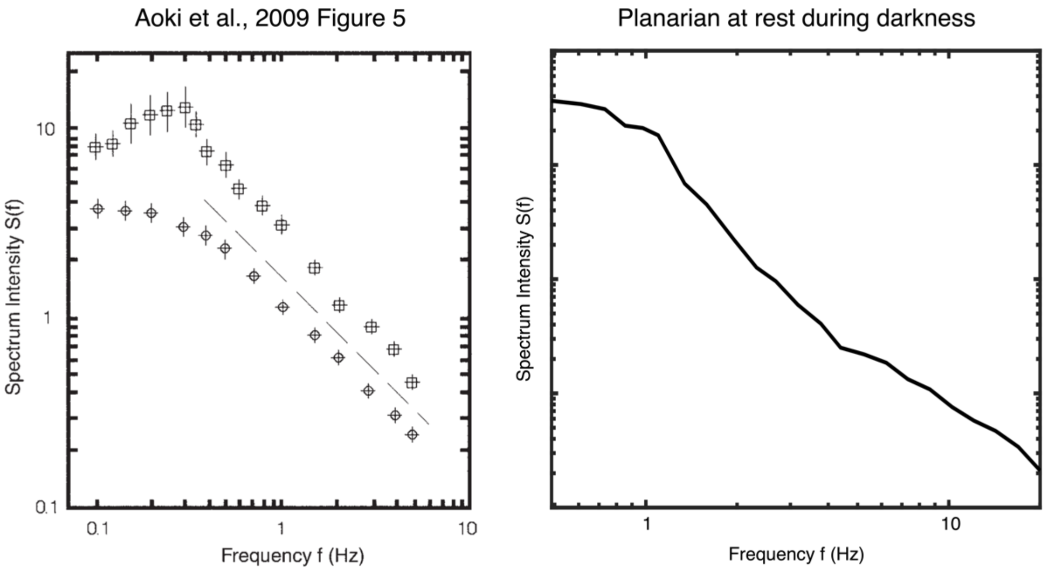

Figure A2: Direct comparison between the power spectra obtained by Aoki and colleagues (left) and from the surface EEG recordings at rest during darkness (right). In the left plot, the dashed line indicates an exponent x = 1, circles denote initial recordings in an active state and squares denote recordings after recovery from anesthesia. Please note the different amplitude and frequency scales. Reproduced with permission from Aoki et al., 2009.
